# Relation to enterocins and herbal extracts of fecal hemolytic *Escherichia coli* from domestic ducks detected with MALDI-TOF mass spectrometry

**DOI:** 10.3382/ps/pez371

**Published:** 2019-07-12

**Authors:** J Ščerbová, A Kandričáková, Ľ Hamarová, A Lauková

**Affiliations:** Institute of Animal Physiology, Centre of Biosciences of the Slovak Academy of Sciences, Šoltésovej 4–6, 040 01 Košice, Slovakia

**Keywords:** *Escherichia coli*, ducks, susceptibility, resistance, antimicrobials

## Abstract

Surveillance studies have generally reported an increase in *Escherichia coli* strains resistant to major classes of antibiotics used for animals’ treatment. The aim of this study was to test the susceptibility of 25 strains (isolated from 30 domestic Mallard ducks—*Anas platyrhynchos*, both sex, aged 8 to 14 wk, taxonomically alloted to the species *E. coli* using MALDI TOF mass spectrometry system) to antimicrobials (antibiotics, enterocins, and herbal extracts). Testing was performed using the agar disc method and the agar diffusion method. A total of 19 *E. coli* strains were multiresistant to antibiotics; but 10 of those strains were susceptible to enterocins with an inhibition activity of 100 AU/mL. All strains were susceptible to herbal extracts. These results indicate further benefit application of enterocins and herbal extracts to prevent/reduce problems caused with *E. coli.* Moreover, additional studies are in process.

## INTRODUCTION

Poultry products can be source of foodborne pathogens and/or source of antimicrobial-resistant bacteria (Marshall and Levy, 2011). Their presence can result from selection pressure on bacteria due to the indiscriminate use of antimicrobials in aviculture as feed additives or as therapy. *Escherichia coli* is an incredibly diverse bacterial species with the ability to colonize and persist e.g., in environment and warm-blooded animal hosts, including poultry (Yan and Polk, 2004). Some strains can be, however, pathogenic and causing illnesses. They are categorized as either diarrheagenic *E. coli* or extraintestinal pathogenic *E. coli* (Kunert Filho et al., 2015). Bacteria can be intrinsically resistant to antibiotics, but they can also acquire resistance to antibiotics via mutations in chromosomal genes and by horizontal gene transfer. Bacterial strains carrying mobile genetic elements (plasmids, transposons, and integrons) can play important role in the dissemination of transmissible resistance genes. Surveillance studies have generally reported an increase in resistant *E. coli* strains occurrence, especially resistance to antibiotics used for the treatment of livestock and companion animals. Ducks can also be source of multiresistant *E. coli* (Blair et al., 2015). Increases in antimicrobial-resistant bacteria have generated significant concern on food safety. Animal-derived food can be contaminated e.g., by those multiresistant *E. coli* during slaughtering (Marshall and Levy, 2011; Asai et al., 2014). Therefore, food animals and their living environments are reservoirs of both resistant bacteria and resistant genes that could be transferred to human either by direct contact between animals and human or via the food production chain (Marshall and Levy, 2011). From this reason, farmers have interest in natural substances to prevent antibiotic resistance increase. Many Gram-positive bacteria, including some representatives of the phylum Firmicutes can be a source of bacteriocins (Franz et al., 2011). Bacteriocins are prokaryotic proteins or peptides with antimicrobial activity. Among them enterocins are bacteriocins produced mostly by enterococci, belonging to class II bacteriocins, which are thermostable with a broad antimicrobial spectrum (Franz et al., 2011; Simonová and Lauková, 2007; Cotter et al., 2013). Nowadays, there are many studies reporting inhibition activity of enterocins in animals (Lauková et al., 2004; Szabóová et al., 2008). Similarly, phytoadditives, especially oregano or sage, have been shown to present inhibiton activity in vitro as well as in vivo (Szabóová et al., 2013). Both Oregano (*Origanum vulgare*) and sage (*Salvia officinalis*) belonged to the family Lamiacae. Both additives were found effective in reduction of clostridiae or pseudomonads in broiler rabbits. Moreover, immuno-stimulative effect was also demonstrated (Szabóová et al., 2012a,b). The aim of our study was to test fecal hemolytic *E. coli* from domestic ducks in relation to antimicrobials especially enterocins and herbal extracts (sage, oregano) to indicate their possible use to prevent bacterial contamination in domestic breeding and/or during slaughtering and meat processing as well.

## MATERIAL AND METHODS

### Study Design

A total of 22 mixtures of feces (n = 22) from 30 domestic Mallard ducks (*Anas platyrhynchos*), both sex, aged 8 to 14 wk, and clinically healthy, were sampled in different private breeders. They were sampled continually; first sampling includes 11 animals (8 mixtures); second sampling comprises 10 animals (7 mixtures) and third one sampling involved 9 ducks (7 mixtures). Ducks have been bred with straw litter. They have had grassed run and access to the waterfront. The diet consisted of ground barley, nettles, and abundant grazing which amounted to 50% of the feed. The animals had access to water ad libitum. The handling with animals followed rules of Ethic commision, and it was approved by Slovak Veterinary and Food Administration. Fresh feces were sampled in the duck coop by hand using gloves, immediately after being voided by the birds to prevent other contamination. Subsequently, fecal samples were put into sterile packs placed into a transport fridge and driven to our laboratory.

### Identification of Isolates Using MALDI-TOF Mass Spectrometry, Phenotypization and Hemolysis Testing

Samples were treated according to the standard microbiological method (International Organisation for Standardization, ISO); 1 g of mixture feces was diluted in 9 mL of Ringer solution (pH 7; Merck, Darmstadt, Germany). Samples were stirred using a Stomacher–Masticator (Spain) and appropriate dilutions were plated onto Mac Conkey agar (Oxoid, United Kingdom) to detect *E. coli.* Plates were incubated at 37°C for 24 h. The bacterial counts were expressed in colony forming unit per gram of feces (log_10_ CFU/g ± SD). Randomly picked up colonies were checked for purity and submitted for further analysis. Phenotypization was performed using the BBL Crystal Enteric/Nonfermenter ID System (Becton and Dickinson, Cockeysville). Strains were prepared according to the producer's instructions. Evaluation was carried out with BBL Crystal Mind software (Becton and Dickinson). *Escherichia coli* ATCC 25922 was control strain. Subsequently, strains were identified using the MALDI-TOF mass spectrometry (**MS**) based on protein “fingerprints” (Alatoom et al., 2011, MALDI-TOF MS Bruker Daltonics). A single colony from Mac Conkey agar (Oxoid) was mixed with matrix (α-cyano-4-hydroxycinnamic acid and trifluoroacetic acid). Suspension was spotted onto a MALDI plate and ionized by nitrogen laser (wavelength 337 nm, frequency 20 Hz). Results were evaluated using the MALDI-TOF MS Biotyper 3.0 (Bruker Daltonics) identification database. Taxonomic allotment was evaluated on the basis of highly probable species identification (value score 2.300 to 3.000) and secure genus identification/probable species identification (2.000 to 2.299). Control strains were those involved in the identification system. Identical colonies evaluated using the MALDI-TOF MS score value were excluded. The phylogenetic tree was constructed using the neighbor-joining method with the software MEGA5 (Tamura et al., 2011).

Identified strains were checked to form hemolysis (phenotype) on Trypticase soy agar (**TSA**, Becton and Dickinson) supplementd with 5% of defibrinated sheep blood. Inoculated media were incubated at 37°C for 24 to 48 h under aerobic conditions. Hemolytic phenotype was evaluated demonstrating type of clearing zones around the colonies.

### Antibiotic Profile

Antibiotic susceptibility/resistance phenotype was performed according to the Clinical and Laboratory Standards Institute (CLSI, 2013) by the disc diffusion method on Mueller Hinton agar (Oxoid Ltd., Basingstoke, Hampshire, United Kingdom). Antibiotic discs (Becton and Dickinson; Lach-Ner, Czech Republic and Oxoid, United Kingdom) were as follows: penicillin (Pnc 10 IU), chloramphenicol (Chc 30 *µ*g), tetracycline (Tct 30 *µ*g), cefuroxime (Crx 30 *µ*g), amikacin (Ak 30 *µ*g), kanamycin (Kan 30 *µ*g), aztreonam (Atm 30 *µ*g), cephalothin (Kf 30 *µ*g), phosphomycin (Phos 50 *µ*g), cinoxacin (Cin 100 *µ*g), cefepime (Fep 30 *µ*g), ciprofloxacin (Cip 5 *µ*g), and gentamicin (Cn 120 *µ*g). Plates were cultivated at 37°C for 18 h. Evaluation of susceptibility/resistance was performed according to the manufacturer´s instruction. Size of inhibition zones was expressed in milimeter. For 2 antibiotics, cefotaxime (CTX 0.002 to 32 *µ*g/mL) and ciprofloxacin (CIP 0.002 to 32 *µ*g/mL), the antimicrobial gradient method using strips was used (BioMérieux, Marcy-l'Etoile, Etest, France) because discs were not disposable. Antibiotic strips were placed on the inoculated Mueller-Hinton agar (Oxoid) and incubated overnight. After incubation, strains were evaluated as susceptible/resistant according to EUCAST breakpoint table (The European Committee on Antimicrobial Susceptibility Testing, 2017). *Escherichia coli* ATCC 25922 was used as control strain in both methods.

### Susceptibility to Enterocins and Herbal Extracts of Identified *E. coli*

Identified *E. coli* were treated with partially purified bacteriocins (**PPBs**, enterocins; dose 10 *µ*L of each) using the agar spot method (De Vuyst et al., 1996). Briefly, TSA plates (1.5% agar, Becton and Dickinson) were overlaid with 0.7% TSA containing 200 *µ*L of tested *E. coli* broth culture. Natural substances (10 *µ*L) were dropped on the surface of 0.7% agar layer and incubated at 37°C. Enterocins used are listed in Table 1. They were characterized and prepared at our Laboratory of Animal Microbiology (Košice, Slovakia). The inhibition activity of PPBs was defined as the reciprocal of the highest dilution producing a distinct inhibition of an inhibition lawn, and it was expressed in arbitrary unit per mililiter of culture medium (AU/mL). The most susceptible indicator strain *Enterococcus faecium* EA 5 (isolated from piglet) was used as control strain. Activity of PPBs against the most susceptible indicator (EA5) reached 3.200 up to 51.200 AU/mL (Table 1).

**Table 1.** Partially purrified bacteriocins (PPBs) and their producing strains.

PPBs of enterocins	Producer strain	PPBs preparation
Ent EM 41	*E. faecium* EM 41	Lauková et al. (2012)
Ent A(P)	*E. faecium* EK 13 = CCM 7419	Mareková et al. (2003)
Ent M	*E. faecium* AL 41 = CCM 8558	Mareková et al. (2007)
Ent 4231	*E. faecium* EF1 = CCM 4231	Lauková et al. (1993)
Ent 9296	*E. faecium* 9296	Marciňáková et al. (2008)
Ent 412	*E. faecium* EF 412	Lauková et al. (2008)
Ent 55	*E. faecium* EF 55	Strompfová and Lauková (2007)
Ent 131	*E. faecium* Hč 13/1	unpublished data

Activity of enterocins against the principal indicator *Enterococcus avium* EA5: Ent 55–51 200 AU/mL, Ent EM41, Ent 412, Ent 9296, Ent A(P), Ent 131–25 600 AU/mL, EntM-6400 U/mL, Ent 4231–3200 AU/mL.

Moreover, susceptibility of identified *E. coli* to oregano and sage extracts (10 *µ*L of both extracts, Calendula a.s., Nová Ľubovňa, Slovakia) was tested using the qualitative agar diffusion test and it was expressed in inhibion size zones (mm). Extract of oregano contained carvacrol 55 ± 3% (gas chromatography analysis; density:0.959 ± 0.002 g/cm^3^; refractive index:1.515 ± 0.001, Szabóová et al., 2012b). *Salvia officinalis* extract contained 24% of thujone, 18% of borneol, and 15% of cineole (Szabóová et al., 2012a).

## RESULTS

**Figure 1.**
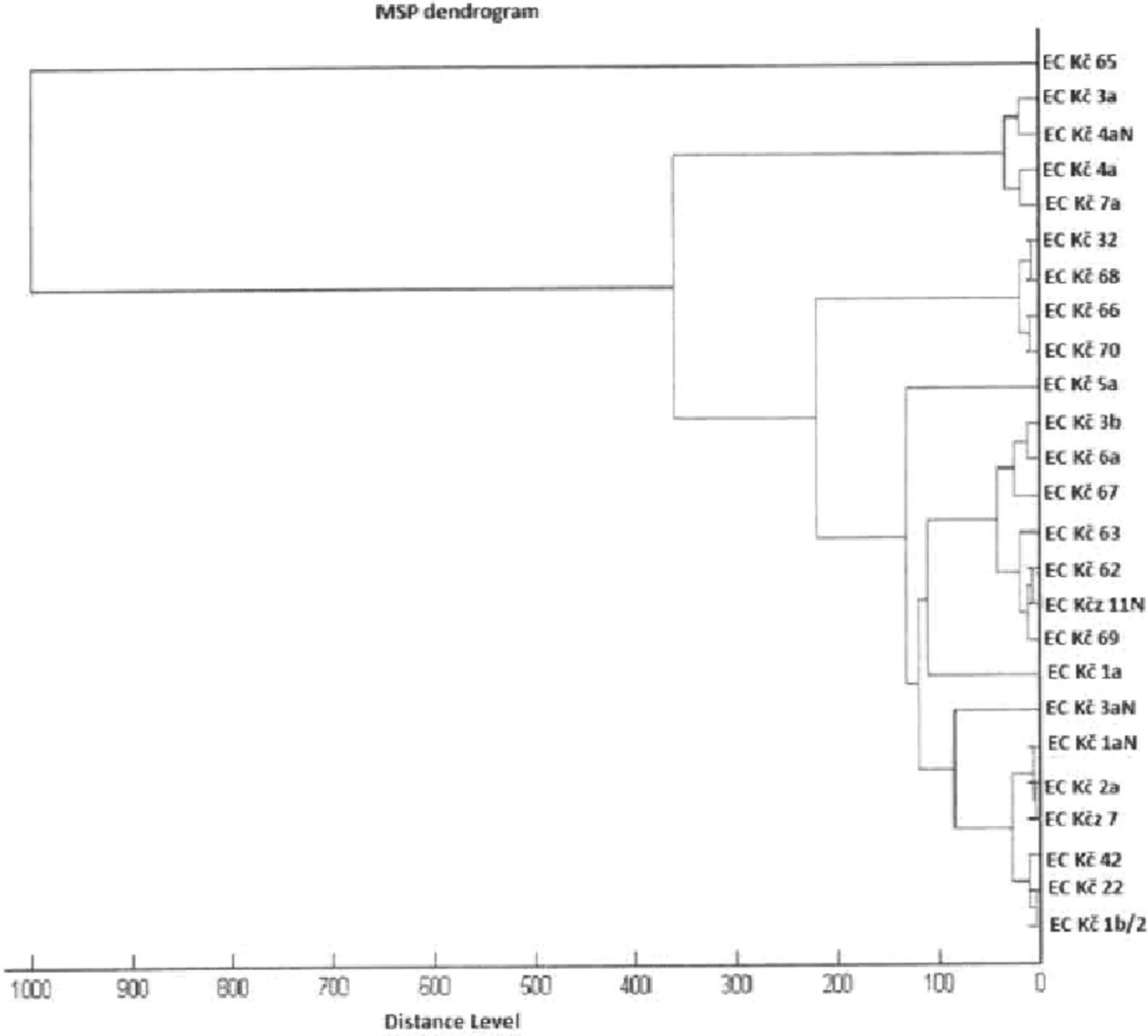
Dendrogram of identified *Escherichia coli* strains from ducks.

The average count of presumptive *E. coli* was 4.6 ± 0.7 log10 CFU/g. A total of 25 strains were identified and belonged to the species *E. coli* by evaluation of protein spectra (MALDI-TOF MS system). Identification score values of 9 identified strains ranged from 2.300 to 3.000 (Ec Kč 2a, Ec Kč 22, Ec Kč 32, Ec Kč 5/A, Ec Kč 6a, Ec Kč 62, Ec Kč 66, Ec Kč 67, Ec Kč 68); other 16 strains were evaluated with score value in range from 2.000 to 2.299 (Figure 1). In addition, phenotypic properties confirmed allotment of 25 strains to the species *E. coli* showing the same reaction (e.g., for disacharides or enzymes) as the control strain *E. coli* ATCC 25922. Of 25, 24 *E. coli* tested formed *ß*-hemolysis (Tables 2 and 3).

**Table 2.** Hemolytic activity, antibiotic profile, and susceptibility to enterocins of *Escherichia coli* from ducks.

	Antibiotics										Herbal extracts										
Strains	CHC	TCT	CN	AK	ATM	CRX	KF	FEP	CIN	CIP	Sage	Oregano	Hemolysis	Ent EM 41	Ent 131	Ent M	Ent 55	Ent A(P)	Ent 9296	Ent 412	Ent 4231
Ec Kč 1a	R	R	R	R	R	R	R	R	S/20	I	+	+	+	–	–	–	–	–	–	–	–
Ec Kč 1b/2	R	R	S/17	R	R	R	R	R	S/25	S/25	+	+	+	–	–	–	–	–	–	–	–
Ec Kč 2a	S/20	R	I	R	R	R	R	R	I	S/21	+	+	+	–	100	–	–	–	–	–	–
Ec Kč 22	S/20	R	S/15	R	R	R	R	R	S/19	S/23	+	+	+	–	–	–	–	100	–	–	–
Ec Kč 3a	S/28	R	S/17	R	R	R	R	R	S/21	I	+	+	+	100	100	–	100	–	–	–	–
Ec Kč 32	R	R	S/16	R	R	R	R	R	S/25	S/21	+	+	+	–	–	–	–	–	–	–	–
Ec Kč 3b	S/25	R	S/19	I	S/22	I	S/19	S/23	S/25	S/25	+	+	+	–	–	–	–	–	–	–	–
Ec Kč 42	S/22	R	S/29	R	R	R	R	R	I	S/24	+	+	+	–	100	–	–	100	–	–	–
Ec Kč 4a	S/28	R	S/25	R	R	R	R	R	S/28	S/22	+	+	+	–	–	–	–	–	–	–	–
Ec Kč 5/A	S/21	R	S/20	R	I	I	R	R	S/24	R	+	+	+	–	–	–	–	–	–	–	–
Ec Kč 6a	R	R	S/24	R	R	R	R	R	S/21	R	+	+	+	–	–	–	–	–	–	–	–
Ec Kč 7a	S/20	R	S/16	R	I	S/20	R	R	S/25	R	+	+	+	100	–	–	–	–	–	–	–
Ec Kč 62	S/20	R	S/15	R	I	I	R	R	S/23	I	+	+	+	–	–	–	–	–	–	–	–
Ec Kč 63	S/21	R	S/16	S/17	I	I	I	S/22	S/25	I	+	+	-	–	–	–	–	–	–	–	–
Ec Kč 65	S/21	I	S/18	R	S/23	R	R	R	S/23	S/22	+	+	+	–	–	–	–	–	–	–	100
Ec Kč 66	S/20	R	S/16	R	S/23	R	I	R	S/25	I	+	+	+	–	–	–	100	–	–	–	–

All strains were susceptible to phosphomycine (Phos; inhibition zones from 17 to 30); all strains were resistant to penicillin (Pnc) and kanamycin (Kan); ATB—antibiotics, Ec—*Escherichia coli*; Ent—enterocin; CHC—chloramphenicol, Tct—tetracycline, CN—gentamicin, AK—amikacin, ATM—aztreonam, CRX—cefuroxime, KF—cefalotin, FEP—cefepime, CIN—cinoxacin, CIP—ciprofloxacin; + hemolysis (phenotype) or herbal extract inhibit the growth of tested strains; –enterocins did not inhibit the growth of tested strains; R—resistance; e.g., S/21 means a size of inhibition zone in millimeter; I-intermediate reaction; activity of enterocins is expressed in arbitrary units per milliliter.

**Table 3.** Hemolytic activity, antibiotic profile, and susceptibility to enterocins of *Escherichia coli* from ducks.

	Antibiotics										Herbal extracts										
Strains	CHC	TCT	CN	AK	ATM	CRX	KF	FEP	CIN	CIP	Sage	Oregano	Hemolysis	Ent EM 41	Ent 131	Ent M	Ent 55	Ent A(P)	Ent 9296	Ent 412	Ent 4231
Ec Kč 67	S/19	R	S/17	R	S/20	S/18	R	S/20	S/22	I	+	+	+	–	–	–	–	–	–	–	–
Ec Kč 68	S/21	R	S/18	S/18	S/23	R	R	S/20	S/25	I	+	+	+	–	–	–	100	–	–	–	–
Ec Kč 69	S/20	R	S/16	R	I	R	R	R	S/21	I	+	+	+	–	–	–	–	–	–	–	–
Ec Kč 70	S/22	S/20	S/18	R	S/25	I	S/19	S/19	S/26	I	+	+	+	–	–	–	–	–	–	–	–
Ec Kč 1aN	S/22	R	S/19	S/18	S/29	I	I	S/23	I	I	+	+	+	–	–	–	–	–	–	–	–
Ec Kč 11N	S/20	R	Int.	R	R	R	R	R	S/24	S/21	+	+	+	–	100	–	–	100	–	–	–
Ec Kčz 7	S/20	R	S/20	R	S/21	I	R	R	S/25	S/25	+	+	+	100	–	–	100	–	–	–	–
Ec Kč 3aN	S/20	R	S/15	R	R	R	R	R	S/25	I	+	+	+	–	–	–	–	–	–	–	–
Ec Kč 4aN	S/20	R	S/15	R	R	R	R	R	S/23	I	+	+	+	–	–	–	–	–	–	–	–

All strains were susceptible to phosphomycine (Phos; inhibition zones from 17 to 30 mm); all strains were resistant to penicillin (Pnc) and kanamycin (Kan); ATB—antibiotics, Ec—*Escherichia coli*; Ent—enterocin; CHC—chloramphenicol, Tct—tetracycline, CN—gentamicin, AK—amikacin, ATM—aztreonam, CRX—cefuroxime, KF—cefalotin, FEP—cefepime, CIN—cinoxacin, CIP—ciprofloxacin; + hemolysis (phenotype) or herbal extract inhibit the growth of tested strains; –enterocins did not inhibit the growth of tested strains; R—resistance; e.g., S/21 means a size of inhibition zone in millimeter; I—intermediate reaction; activity of enterocins is expressed in arbitrary units per milliliter.

### Susceptibility Testing of *E. coli* to Antibiotics, Enterocins, and Herbal Extracts

Identified *E. coli* showed 100% of resistance to penicillin and kanamycin. Tetracycline resistance (92%) and 84% of amikacin resistance were noted in tested strains. Strains were mostly resistant to cephalosporines; resistance to cefepime and cephalotin was of 76 to 80%; 16 strains were resistant to cefuroxime (64%). Resistance to quinolone antibiotic ciprofloxacin was low (16%). Strains *E. coli* Kč 1a and *E. coli* Kč 6a were resistant to 10 antibiotics; *E. coli* Kč 1b/2 and *E. coli* Kč 32 were resistant to 9 antibiotics. All strains were susceptible by intermediate reaction to cinoxacin (Tables 2 and 3). Approximately 32% of *E. coli* were resistant to cefotaxim and 56% of tested *E. coli* were resistant to ciprofloxacin.

Among 25 tested *E. coli*, 10 strains were susceptible at least to one enterocin. Four strains of *E. coli* were susceptible to enterocins Ent 131 and Ent 55. Three strains—Ec Kč 22, Ec Kč 42, Ec Kč 11 N; (Tables 2 and 3) were susceptible to Ent A(P) and Ent EM 41. On the other hand, 15 strains were resistant to enterocins. Multiresistant strain Ec Kč3 was susceptible to enterocins; its growth was inhibited by Ent EM 41, Ent 131, and Ent 55. Strains Ec Kč 42 and Ec Kčz 7 were susceptible to 2 Ents. Of 25, 7 strains were susceptible to one enterocin (Tables 2 and 3). Growth of tested strains was inhibited with inhibition activity of 100 AU/mL.

*Escherichia coli* isolated from ducks were susceptible to oregano and sage. Inhibition zones using oregano were bigger (up to 30 mm); after sage treatment, the inhibition zones measured up to 20 mm on average).

## DISCUSSION

*Escherichia coli* of animal origin are increasingly associated with extraintestinal diseases in human. The aim of the research is increasingly common in avian *E. coli*; avian *E. coli* can cause diseases not only in poultry, but are often associated with extraintestinal *E. coli* in human causing mainly infection of urinary tract (Obeng et al., 2012). In some cases, human treatment is limited due to a high percentage of antimicrobial resistance (Laxminarayan et al., 2013). In our study, the average count of *E. coli* in ducks’ feces was 4.6 ± 0.7 log10 CFU/g. Some authors presented occurrence of *E. coli* in poultry in count 7.7 to 9.9 log10 CFU/g (Murphy et al., 2005). Twenty-five strains were taxonomically alloted to the species *E. coli* using the MALDI-TOF MS system. Although, 20 strains—in pairs (Ec Kč 3a—Ec Kč 4aN, Ec Kč 4a—Ec Kč 7a, Ec Kč 32–Ec Kč 68, Ec Kč 66–Ec Kč 70, Ec Kč 3b—Ec Kč 6a, Ec Kč 62–Ec Kčz 11 N, Ec Kčz 11 N—Ec Kč 69, Ec Kčz 1aN—Ec Kč 2a, Ec Kč 2a—Ec Kčz 7, Ec Kč 22–Ec Kč 1b/2, Figure 1) were identical on the basis of phylogenetic analysis, they differ in susceptibility to enterocins or to antibiotics.

*Escherichia coli* from ducks showed mostly antibiotic resistance; even multiresistant strains were determined. A high number of multiresistant *E. coli* isolated from either meat or intestinal contents have also been reported by other authors (Adelowo et al., 2014; Koga et al., 2015). According to Hammerum and Heuer (2009), critically important antimicrobial agents for *E. coli* are cephalosporines (mainly III and IV generation), quinolones, sulfonamides, and aminoglycosides. However, using cephalosporin antibiotics such as cefotaxime or quinolone antibiotics such as ciprofloxacin, a high number of *E. coli* strains become susceptible to enterocins. Among aminoglycosides, 4% resistance to gentamicin, 100% resistance to kanamycin, and 84% resistance to amikacin was recorded. Tested *E. coli* were also mostly resistant to tetracycline (92%), penicillin (100%), and to other cephalosporines (cefuroxim, cefalotin, and cefepime). High percentage of resistance to tetracycline and penicillin in *E. coli* was also presented by Akond et al. (2009). In our study, 19 strains (76%) were multiresistant. This significant multiresistance in domestic breeding is striking, since no medication was applied to the breed. It could be probably explained with the fact that streams’ water (to which ducks are occasionally fed) can be a source of possible antibiotics’ transfer in water environment. Another possible condition is their free movement in the large area where they can come into contact with other domestic animals which can be a source of resistant bacterial strains. The significant increase of antibiotic-resistant bacteria and residua in meat products has evoked and has supported the research on new natural originated antimicrobials (enterocins, herbal extracts, etc.). Antimicrobial activity of enterocins has been confirmed in our previous in vitro and in vivo studies (Lauková et al., 1999, 2015; Ščerbová and Lauková 2016a,b). Moreover, tested trains were more susceptible to enterocins and herbal extracts than to antibiotics. A total of 19 *E. coli* strains were recorded as multiresistant to antibiotics, but 10 of those strains were susceptible to enterocins and all strains were susceptible to herbal extracts. Although enterocins used are known to have a broad inhibition spectrum (Lauková et al., 1993, 2008, 2012; Mareková et al., 2003, 2007; Strompfová and Lauková, 2007; Marciňáková et al., 2008), to explain why 10 strains were susceptible to some Ents and to another ones not could be probably because of phenomenon of immunity in class II bacteriocins (in which used Ents belong). One gene encodes for the immunity protein, usually a basic protein between 50 and 150 amino acid residues long that is loosely associated with the membrane (Cleveland et al., 2001). Effective combinative treatment of bacteriocin-lantibiotic nisin with herbal extract cinnamaldehyde and EDTA to control growth of *E. coli* strains from swine origin was reported by Des Field et al. (2017), which indicates a new approach to eliminate or to prevent *E. coli* agents.

## CONCLUSION

Although identified species of hemolytic *E. coli* were mostly resistant to antibiotics, they were susceptible to enterocins and herbal extracts. This indicates a new approach using application of enterocins and herbal extracts to prevent or reduce disorders caused with *E. coli.*
